# Study of Depression and Its Associated Factors Among Patients of Diabetes Mellitus (DM) and Hypertension (HTN) Attending a Primary Health Center (PHC) in a Rural Area of New Delhi, India

**DOI:** 10.7759/cureus.33826

**Published:** 2023-01-16

**Authors:** Siddharth Sekhri, Anita Verma

**Affiliations:** 1 Community Medicine, Vardhman Mahavir Medical College and Safdarjung Hospital, New Delhi, IND

**Keywords:** mental health, india, primary health centre, rural, non communicable diseases, hypertension, diabetes, depression

## Abstract

Introduction: Depression is among the most common mental disorders which is a leading cause of disability and is a negative prognostic indicator in many non-communicable chronic diseases such as diabetes mellitus (DM) and hypertension (HTN). Depression among these patients can further worsen their disease condition. Existing information on this topic has mostly come from tertiary care hospital setting. Hence, present study was done among patients attending a primary health center (PHC) in New Delhi.

Methods: This was a cross-sectional study conducted among 210 participants having diabetes and/or hypertension attending the non-communicable diseases (NCD) clinic at PHC, Fatehpur Beri, New Delhi. Simple random sampling was done and a pre-designed, semi-structured, interview-based questionnaire was used. Depression was assessed using a Patient Health Questionnaire-9 (PHQ-9). Data were analyzed using SPSS software version 21.0 (IBM Corp., Armonk, NY).

Results: The overall prevalence of depression was found to be 49% (n=103), out of which most had moderate depression (23.8%), 16.2% had moderately severe depression, and 9% had severe depression. Multivariate analysis results revealed the odds of having depression to be significantly higher among those who belonged to lower socioeconomic class (adjusted odds ratio, aOR=2.9, confidence interval, CI=1.2-7.4); had uncontrolled diabetes mellitus/hypertension (DM/HTN) (adjusted odds ratio, aOR=2.5, CI=1.1-6.1); had associated comorbidities (aOR=5.9, CI=2.4-15); sedentary lifestyle (aOR=7.8, CI=2.4-25.1); who had past history of coronavirus disease 2019 (COVID-19) infection (aOR=14.7, CI=5.4-39.6); and those who lost family member(s) due to COVID-19 (aOR=10.1, CI=1.3-79.4).

Conclusion: Prevalence of depression in patients with DM/HTN was found to be significantly high and various factors were found to be significant. Therefore, every such patient should be screened for depression; and periodic follow ups should also be conducted to prevent future complications and improve prognosis of the disease.

## Introduction

Depression (major depressive disorder) is defined by the World Health Organization as ‘a common mental disorder, characterized by sadness, loss of interest or pleasure, feelings of guilt or low self‑worth, disturbed sleep or appetite, feelings of tiredness, and poor concentration’ and is among the most common mental disorders [1]. It is a leading cause of disability and a significant contributor to the overall global burden of disease. Worldwide, it affects more than 264 million people [1]. At its worst, depression can lead to suicide. In India, 45.7 million people were suffering with depression in the year 2017 [2]. Depression is a negative prognostic indicator in many chronic medical diseases. Diabetes mellitus (DM) and hypertension (HTN) are the two most common non-communicable chronic illnesses. Both are shown to serve as a risk factor for depression [3-4]. Depression among these patients can result in poor self-care that can further worsen their disease condition. It leads to lack of adherence to treatment, loss to follow up, and poor compliance to lifestyle modification. It also impairs quality of life and several aspects of the functioning of patients with DM/HTN [4]. However, co-morbid mental disorders are often under-recognized and not always effectively treated. A knowledge about the extent and factors associated with depression in patients with DM/HTN might be of immense importance as it may pave the way for the clinicians toward an improved and effective management of this burdensome disease. Also, discovering depression earlier among patients with DM/HTN can make it easier for them to cope with their condition, leading to better health and quality of life. Depression among patients with DM/HTN attending primary healthcare setting is relatively common, but they are usually diagnosed less [5]. Also, existing information on this topic has mostly come from tertiary care hospital setting and patients attending tertiary care centers are unlikely to be representative of entire disease spectrum. Very few studies have been done in primary health setting. Hence, present study was planned to determine prevalence of depression and associated factors among patients with DM/HTN attending the non-communicable diseases (NCD) clinic in a primary health center (PHC) in Fatehpur Beri, New Delhi.

## Materials and methods

An observational, descriptive cross-sectional study was conducted from January 2021 to December 2021 among participants having DM and/or HTN attending the NCD clinic at a primary health center (PHC), Fatehpur Beri, New Delhi. It caters to nearby five villages and comes under South Delhi Municipal Corporation.

Inclusion criteria

Patients who were diagnosed with DM/HTN or both; having age of 30 years and above.

Sample size

Taking prevalence of depression to be 42.5% in patients of DM and/or HTN attending a health center in urban slum of East Delhi as reported by Taneja et al. [6] in the year 2015, the sample size was calculated by the formula (Zα/2)2 PQ / L2: to be 210 using 95% confidence interval (CI), 7% absolute error and non-response rate of 10% (non-response was taken as those registered patients who could not be contacted even after three attempts till the time of completion of data collection).

Sampling technique

All registered patients in the NCD clinic from 1st January 2019 till 31st December 2020 who were given a registration number were taken as the sampling frame (total number=630 patients). Participants were selected by simple random sampling from it. Computer generated random number software was used for this purpose and the NCD registration numbers selected were chosen as study participants. Participants were telephonically contacted and called to the NCD clinic on a specific day. Help of ASHAs was taken for those who could not be contacted telephonically.

Study tool

A pre-designed, pre-tested, semi-structured, interview-based questionnaire in Hindi language was used. The questionnaire contained questions regarding socio-demographic data including age, sex, marital status, socio-economic status, education, occupation, number of family members, type of family, etc. Questions regarding the disease (DM/HTN), treatment history, and other comorbidities were asked.

Depression was assessed using Patient Health Questionnaire-9 (PHQ-9). It is a previously validated nine-item questionnaire with score ranging from 0 to 27. It assesses the symptoms experienced by participants during the two‑week period before they take the survey. It assesses the presence of major depressive disorder using modified Diagnostic and Statistical Manual fourth edition (DSM-IV) criteria [7]. This score is divided into five categories where score of 0-4 represents none to minimal depression (where no intervention is required); score of 5-9 represents mild depression which also does not require any active treatment and PHQ-9 should be repeated during follow-up after three months for them; score of 10-14 represents moderate depression which requires active treatment and/or pharmacotherapy, score of 15-19 represents moderately severe depression and a score of more than 20 represents severe depression [7]. 

Various studies have demonstrated the PHQ-9 scale having reasonable accuracy in identifying cases of depression among patients with DM/HTN and is a useful screening tool in NCD setting. The optimal cut-off score for major depression was found to be 10 in these studies, with sensitivity in the range of 84%-88% and specificity in the range of 82%-94% [8-9]. 

Examination and anthropometric measurement of the study participants were done -- height was measured by stadiometer and weight was measured using standardized recently calibrated digital weighing machine. Blood pressure was measured by standardized blood pressure measurement procedure using electronic digital blood pressure monitoring device [10] and blood glucose was measured by standardized blood glucose measurement using an electronic blood glucose meter [11].

Operational definitions

1. Diagnosed patient of HTN -- based on at least two measurements taken in the clinic or by a healthcare provider during at least two visits, which were at least 1-4 weeks apart. HTN was diagnosed when the blood pressure was persistently above a systolic of 140 mm Hg and/or diastolic of 90 mmHg [10].

2. Diagnosed patient of DM - symptoms of diabetes (polyuria, polydipsia, weight loss, tiredness, weakness, generalized pruritus, recurrent urogenital infections, delayed healing of wounds, etc.) and random plasma glucose ≥ 200 mg/dL OR fasting plasma glucose ≥ 126 mg/dL; 2 h postprandial glucose ≥ 200 mg/dL; HbA1c ≥ 6.5% [11].

3. Target blood pressure for control of HTN - systolic blood pressure (BP) < 140 mmHg and diastolic BP < 90 mmHg (remains same in diabetics also) [10].

4. Target blood glucose for control of DM - fasting plasma glucose < 125 mg/dL ; 2 h postprandial glucose < 180 mg/dL; HbA1c < 8% [11].

5. A patient was termed as ‘regular in taking medications' when -- he/she took medication every day in appropriate dose, frequency, and time as prescribed by the doctor and never forgot to take medicines in last one month.

6. Physical activity -- as defined by WHO for people living with chronic conditions (DM/HTN, etc.) [12]:

a) Sedentary: less than 150 min of moderate-intensity aerobic physical activity or less than 75 min of vigorous-intensity aerobic physical activity throughout the week.

b) Moderate: should do at least 150-300 min of moderate-intensity aerobic physical activity; or at least 75-150 min of vigorous-intensity aerobic physical activity; or an equivalent combination of moderate- and vigorous-intensity activity throughout the week.

c) Vigorous: moderate-intensity aerobic physical activity to more than 300 min; or do more than 150 min of vigorous-intensity aerobic physical activity; or an equivalent combination of moderate- and vigorous-intensity activity throughout the week.

Outcome measures 

Study participants with PHQ-9 score of more than 10 were considered as suffering from depression [7] and were referred to the Department of Psychiatry, Vardhman Mahavir Medical College & Safdarjung Hospital, New Delhi for further evaluation. Others who had a score of 5 or above were counseled and asked to follow up for repeat screening of depression in the NCD clinic.

Data analysis

Data entry was done in Microsoft Excel spreadsheets using variable coding. Data analysis was done using licensed SPSS software version 21.0 (IBM Corp., Armonk, NY). Data were presented in the form of tables and appropriate diagrams. Qualitative data were summarized as proportions while quantitative data as mean, median, and appropriate measures of dispersion including confidence intervals. Quantitative data was analyzed using t-test and qualitative data by Chi-square/Fisher’s exact test. A p-value less than 0.05 was considered to be significant. 

Ethical consideration

Ethical clearance for the study was obtained from Institutional Review Board (IRB) & Institute Ethical Committee of Vardhman Mahavir Medical College & Safdarjung Hospital, New Delhi (Approval Number: IEC/VMMC/SJH/Thesis/2020-11/CC-82). Each eligible subject was explicitly explained about the purpose of the study by the investigator and an informed written consent was obtained, prior to inclusion. Privacy of participants and confidentiality of information were maintained.

## Results

Table 1 shows that the mean age of the participants was 54.1 years (standard deviation, SD ± 11.9). Maximum participants were in the age group of 50-59 years (30%). More than half of the participants were females (56.2%). Majority of the participants were Hindu by religion (92.9%). More than three-fourth of the participants were married (81.9%) and 16.7% were widows or widowers. Some 39% of the participants were illiterate. More than half of the participants were living in nuclear families (61%) and others in joint family (39%). Almost half of the study participants were homemakers (46.2%) and 17.1% were unemployed.

**Table 1:** Distribution of study participants according to socio-demographic characteristics (N=210). *Modified B.G. Prasad classification, 2021 [13],  ^#^W.H.O. Asian BMI Classification [14]

Characteristics	Numbers (N) (%)
1. Age Group (Years)^	
30-39	28 (13.3)
40-49	39 (18.6)
50-59	63 (30)
60-69	55 (26.2)
70-79	25(11.9)
^Mean age = 54.1 years; S.D. = ±11.9; Max = 78; Min = 30; Range = 48	
2. Gender	
Male	92 (43.8)
Female	118 (56.2)
3. Religion	
Hinduism	195 (92.9)
Islam	15 (7.1)
4. Marital Status	
Unmarried	1 (0.4)
Married	172 (81.9)
Separated	2 (1)
Widowed/widower	35 (16.7)
5. Education	
Illiterate	82 (39)
Primary School	36 (17.1)
Middle School	63 (30)
High School	17 (8.2)
Intermediate	3 (1.4)
Graduate	9 (4.3)
6. Type of Family	
Nuclear	128 (61)
Joint	82 (39)
7. Socio-economic Class*	
Upper (I)	10 (4.8)
Upper middle (II)	25 (11.9)
Middle (III)	41 (19.5)
Lower middle (IV)	104 (49.5)
Lower(V)	30 (14.3)
8. Body Mass Index^#^	
Underweight (<18.5)	3 (1.4)
Normal (18.5-22.9)	80 (38.1)
Overweight (23-24.9)	78 (37.1)
Obese (≥25)	49 (23.4)
9. Disease	
Only Diabetes	80 (38.1)
Only Hypertension	28 (13.3)
Diabetes with Hypertension	102 (48.6)

Almost half of the participants (49.5%) belonged to lower middle socio-economic class, whereas 19.5% participants belonged to middle socio-economic and only 4.8% belonged to upper class category. Majority of the participants (60.5%) had body mass index (BMI) above the normal range, i.e. overweight or obese. Majority of the participants had both diabetes as well as hypertension (48.6%). Some 38.1% participants had DM alone whereas those having HTN alone were 13.3%.

Median duration of disease among both diabetics as well as hypertensives was 3 years (IQR: 1.8). More than one-third had disease in the range of one to five years’ duration (36.3%) and one-third (33%) of the participants had DM/HTN for more than 5 years of duration.

Among the 210 study participants, almost one-third (32.9%) had co-morbidities associated with DM/HTN. Among the participants having comorbidities, more than half of them had arthritis (52.2%). Other comorbidities present were coronary artery disease (18.8%), hypothyroidism (18.8%), bronchial asthma (7.3%), and hepatic steatosis (2.9%).

**Figure 1:**
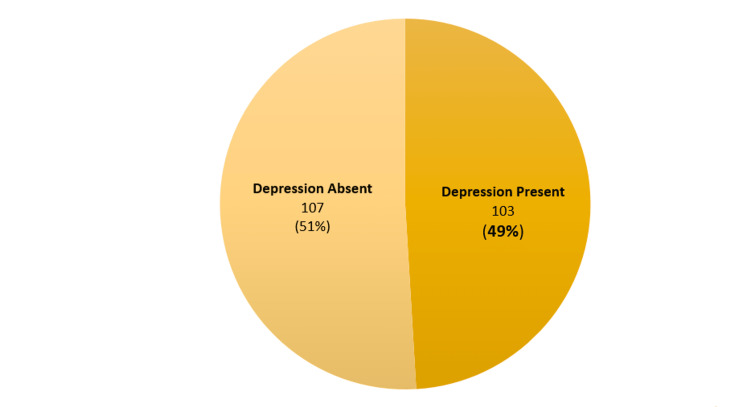
Pie chart depicting distribution of study participants according to prevalence of depression (N=210).

Figure 1 indicates prevalence of depression to be 49% (n=103), i.e. score of 10 or more according to PHQ-9 scale. Whereas, 51% participants did not have depression.

**Figure 2:**
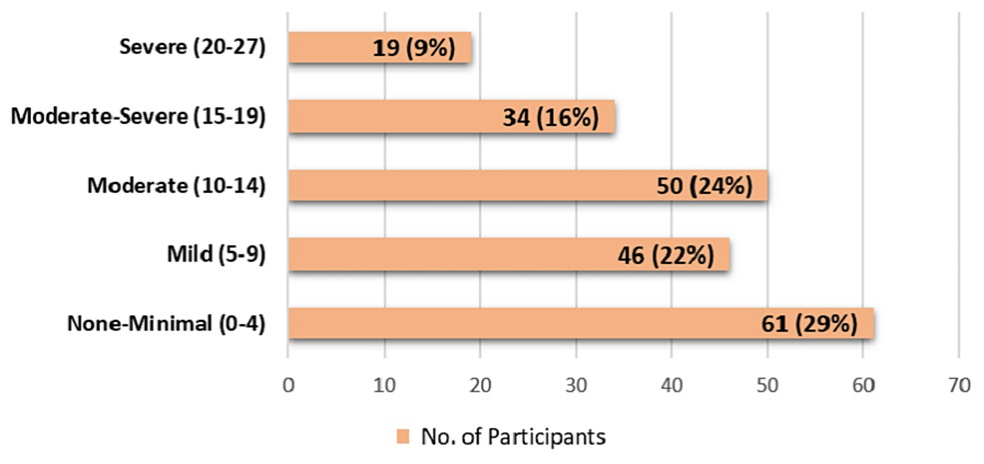
Bar graph depicting distribution of study participants according to severity of depression (N=210).

Figure 2 depicts that 29% participants had none to minimal form of depression, whereas 22% participants had mild form of depression (i.e., PHQ-9 score between five and nine). Out of the 103 participants with depression (i.e., PHQ-9 score of 10 and above), most of them had moderate depression (48.5%); 33% participants had moderately severe type of depression and 18.5% were suffering from severe depression.

Table 2 depicts that, on analyzing various factors with depression among study participants, it was observed that age, female gender, illiteracy, lower socio-economic class, longer duration of DM/HTN, uncontrolled DM/HTN, irregularity in taking medications, presence of comorbidities, sedentary lifestyle, past history of COVID-19 infection, and death of family member(s) due to COVID-19 were found to be significantly associated with depression (p < 0.05). However, religion, marital status, type of family, and BMI of participants had no statistically significant association with depression (p > 0.05).

**Table 2:** Association of socio-demographic characteristics and other factors with depression among study participants (N=210). *(Fischer-exact test), ^#^t-value (t-test)

Characteristic	Depression present n (%)	Depression absent n (%)	Total (%)	χ2	p-value
1. Age					
Mean age ± SD^#^	56.1±11.7	51.9±12.3	54.1±11.9	-2.483^ #^	0.014
Less than 60 years	57(43.8)	73(56.2)	130(100)	3.694	0.054
>= 60 years	46(57.5)	34(42.5)	80(100)		
2. Gender					
Male	38(41.3)	54(58.7)	92(100)	3.928	0.047
Female	65(55.1)	53(44.9)	118(100)		
3. Religion					
Hinduism	93(47.7)	102(52.3)	195(100)	2.007	0.157
Islam	10(66.7)	5(33.3)	15(100)		
4. Marital Status					
Married	86(50.0)	86(50.0)	172(100)	0.345	0.557
Separated/ Widowed/ widower	17(44.7)	21(55.3)	38(100)		
5. Education					
Illiterate or Primary School	69 (58.5)	49(41.5)	118(100)	9.650	0.008
Middle or High School	30 (37.5)	50(62.5)	80(100)		
Intermediate or Graduate	4(33.3)	8(66.7)	12(100)		
6. Socio-economic Class					
Upper (I) or Upper middle (II) or Middle (III)	27(35.5)	49(64.5)	76(100)	8.713	0.003
Lower middle (IV) or Lower(V)	76(56.7)	58(43.3)	134(100)		
7. Family Type					
Nuclear	57(44.5)	71(55.5)	128(100)	2.676	0.102
Joint	46(56.1)	36(43.9)	82(100)		
8. BMI					
Normal (≤22.9)	36 (43.4)	47 (56.6)	83(100)	1.769	0.183
Overweight or Obese (≥23)	67 (52.8)	60 (47.2)	127(100)		
9. Type of Disease					
HTN only	16(57.1)	12(42.9)	28(100)	1.526	0.466
DM only	41(51.2)	39(48.8)	80(100)		
HTN+DM both	46(45.1)	56(54.9)	102(100)		
10. Control of DM/HTN					
Controlled	24 (30.4)	55(69.6)	79(100)	17.660	<0.001
Uncontrolled	79(60.3)	52(39.7)	131(100)		
11. Duration of Disease (in years) (Mean ± SD)^#^					
(a) Diabetes	5.90± 6.69	4.49± 4.42		1.693^#^	<0.05
(b) Hypertension	5.69 ±6.13	4.72± 4.56		1.03^#^	<0.05
12. Regularity in Taking Medication					
Regular	62 (43.4)	81(56.6)	141(100)	5.808	0.016
Not Regular	41(61.2)	26(38.8)	69(100)		
13. Associated Co-morbidities					
Present	54(78.3)	15(21.7)	69(100)	35.093	<0.001
Absent	49(34.8)	92(65.2)	141(100)		
14. Physical Activity					
Moderate/Vigorous	8 (16.3)	41(83.7)	49(100)	27.382	<0.001
Sedentary	95(59.0)	66(41.0)	161(100)		
15. History of COVID-19 Infection					
Present	66 (88.0)	9(12.0)	75(100)	70.832	<0.001
Absent	37 (27.4)	98(72.6)	135(100)		
16. Death of Family Member(s) due to COVID-19*					
Yes	15 (88.2)	2(11.8)	17(100)		<0.001
No	88 (45.6)	105 (54.4)	193(100)		

A logistic regression analysis was performed for factors independently associated with depression by chi-square test to determine independent determinants of depression among patients with DM/HTN. Significantly associated variables identified in univariate analysis with p > 0.2 were included in the model. Multivariate analysis results revealed the odds of having depression to be significantly higher among those who belonged to lower socio-economic class (adjusted odds ratio, aOR=2.9, confidence interval, CI=1.2-7.4); who had uncontrolled DM/HTN (aOR=2.5, CI=1.1-6.1); had associated comorbidities (aOR= 5.9, CI=2.4-15); had sedentary lifestyle (aOR=7.8, CI=2.4-25.1); who had past history of COVID-19 infection (aOR=14.7, CI=5.4-39.6); and those who lost family member(s) due to COVID-19 (aOR=10.1, CI=1.3-79.4). However, age, gender, religion, level of education, family type, BMI, duration of disease, and regularity in taking medication did not show any significant increase in the odds of having depression respectively (Table 3).

**Table 3:** Bivariate and multivariate logistic regression analysis output for factors associated with depression (N=210). OR, odds ratio; CI, confidence interval

Characteristic	Total N (%)	Unadjusted OR; 95%CI	p-value	Adjusted OR; 95%CI	p-value
1. Age					
Less than 60 years	130(61.9)	Reference		Reference	
>= 60 years	80(38.1)	1.7(0.9-3)	0.054	1.3(0.5-3.4)	0.57
2. Gender					
Male	92(43.8)	Reference		Reference	
Female	118(56.2)	1.7(1.01-3)	0.047	1.2(0.4-3.3)	0.67
3. Religion					
Hinduism	195(92.9)	Reference		Reference	
Islam	15(7.1)	2.2(0.7-6.6)	0.157	1(0.2-5.4)	0.99
4. Education					
Illiterate or Primary School	118(56.2)	Reference		Reference	
Middle or High School	80(38.1)	0.4(0.2-0.7)	<0.01	0.8(0.3-2.1)	0.67
Intermediate or Graduate	12(5.7)	0.3(0.1-1.2)	0.1	0.1(0.01-1.9)	0.15
5. Socio-economic Class					
Upper (I) or Upper middle (II) or Middle (III)	76(36.2)	Reference		Reference	
Lower middle (IV) or Lower(V)	134(63.8)	2.3(1.3-4.2)	0.003	2.9(1.2-7.4)	0.01
6. Family Type					
Nuclear	128(61)	Reference		Reference	
Joint	82(39)	1.6(0.9-2.7)	0.102	0.9(0.3-2.2)	0.79
7. BMI					
Normal (≤22.9)	83(39.5)	Reference		Reference	
Overweight or Obese (≥23)	127(60.5)	1.4(0.8-2.5)	0.183	1.2(0.5-2.9)	0.66
8. Control of DM/HTN					
Controlled	79(37.6)	Reference		Reference	
Uncontrolled	131(62.4)	3.5(1.9-6.3)	<0.001	2.5(1.1-6.1)	0.03
9. Duration of Disease (in years)					
(Mean ± S.D.)	5.3±5.5	-	<0.05	1(0.9-1.1)	0.86
10. Regularity in taking Medication					
Regular	141(67.1)	Reference		Reference	
Not Regular	69(32.9)	2(1.1-3.6)	0.01	1.1(0.4-2.7)	0.86
11. Associated Co-morbidities					
Absent	141(67.1)	Reference		Reference	
Present	69(32.9)	6.7(3.4-13.2)	<0.001	5.9(2.4-15)	<0.001
12. Physical Activity					
Moderate/Vigorous	49(23.3)	Reference		Reference	
Sedentary	161(76.7)	7.4(3.2-16.7)	<0.001	7.8(2.4-25.1)	0.001
13. History of COVID -19 Infection					
Absent	135(64.3)	Reference		Reference	
Present	75(35.7)	19.4(8.8-42.9)	<0.001	14.7(5.4-39.6)	<0.001
14. Death of Family Member(s) due to COVID-19					
No	193(91.9)	Reference		Reference	
Yes	17(8.1)	8.9(1.9-40.2)	<0.001	10.1(1.3-79.4)	0.02

## Discussion

Several studies have been conducted to assess the prevalence of depression among patients with either DM or HTN separately and that too mostly in tertiary care hospitals. However, the present study made an effort to evaluate the prevalence in patients having DM or HTN or both of them together attending a PHC in Delhi. A large number of variables that can be associated with depression were taken into account and an attempt to assess their relationship with depression was done in the study.

The present study found that among the 210 study participants, almost half of them were suffering from depression (103; 49%) who had a score of 10 or more according to PHQ-9 scale. This finding is comparable to what Kamble et al. (Haryana; 2021) [15] reported in their study among diabetics and hypertensives, where they found the overall prevalence of depression to be 48.1% taking the same scale and cut-off score as our study. Similar overall prevalence of depression among diabetics and hypertensive patients of 42.6% was reported by Taneja et al. (East Delhi; 2015) [6]. However, studies conducted by Ranjan et al. (Bihar; 2020) [16] and Kulkarni et al. (Mangalore, Karnataka; 2014) [17] found prevalence of depression to be lower than our study (17.1% and 29.1% respectively). This can be due to difference in the study setting as these were done in tertiary health setting. Also, the present study was done during COVID-19 pandemic which itself has been associated with depression and anxiety among the Indian as well as global population and that might have been one of the factors for increased prevalence among the participants [18]. Also, the present study had higher proportion of females as compared to other studies which may be attributed to higher prevalence of depression.

In the present study, 21.9% had mild depression, 23.8% had moderate depression, 16.2% had moderately severe type, and 9% were suffering from severe depression. In the study by Taneja et al. (2015) [6], 36.6% had mild, 34.7% had moderate, and 7.8% had moderately severe depression. None of the participants showed symptoms of severe depression. Khullar et al. (2016) [19] reported 25.3% having mild to moderate depression and 32% having severe depression. These variations may be attributed to difference in the study setting, socio-demographic profile of participants and study period.

In the present study, odds of having depression was found to be significantly higher among those who belonged to lower socio-economic class, similar to what was reported by Lalwani et al. (India; 2019) [20].

Participants whose blood glucose/HbA1c levels or blood pressure were not under control had higher odds of having depression as compared to those who had them under control. In the study by Lalwani et al. (2019) [20] also, it was found that higher proportion of patients with uncontrolled diabetes mellitus (DM) and HTN reported depression (DM: 77.64% vs. 22.36%; HTN: 72.49% vs. 27.51%). Presence of a co-morbidity along with DM and/or HTN was significantly associated with higher depression scores, similar to what was reported by Lalwani et al. (2019) [20]. Present study found depression to be strongly associated with sedentary lifestyle. Similar findings were reported by Khullar et al. [19]. Religion and marital status were not found to have any statistically significant relationship with depression in the current study (p>0.05). Similar findings were reported by Taneja et al. (2015) [6].

Present study found that participants who gave history of COVID-19 infection were at higher odds of having depression as compared to those who did not have the infection in the past. Study by Kaur et al. (India; 2021) [21] also found significant association between the status of COVID-19 infection and depression, but this was done among general population.

This study is not without some limitations. First, because the study was cross-sectional, causal inferences cannot be drawn. Second, since the study was conducted among patients attending NCD clinic in a PHC in Delhi, therefore, the findings may not be generalized to other areas of the country. Also, we cannot comment on the prevalence of depression in the community setting. Few more factors associated with depression could have been assessed, especially among the geriatric population. Lastly, unforeseen prevailing circumstances due to the COVID-19 pandemic during the study duration might have influenced the results.

## Conclusions

The prevalence of depression was high among patients having DM and/or HTN attending NCD clinic of PHC in a rural area of Delhi. Various factors were found to be significantly associated with depression. Hence, it is recommended that every patient with DM and HTN should be screened for depression; and periodic follow ups should also be conducted. This would not only improve DM and HTN-related complications but would also improve quality of life of the patients. Training of healthcare workers (doctors, medical officers, etc.) catering to patients suffering from DM and HTN should be done regarding importance of screening of NCD patients for depression. Also element of counseling regarding importance of maintaining regularity in medication and other healthy lifestyle measures have to be strengthened. Component of mental health can be included in the National Programme for Prevention and Control of Cancer, Diabetes, Cardiovascular Diseases and Stroke (NPCDCS) and in future there is a need to integrate it with National Mental Health Programme.
